# Community-Acquired Pneumonia in Canada During Coronavirus Disease 2019

**DOI:** 10.1093/ofid/ofac043

**Published:** 2022-02-04

**Authors:** Lionel A Mandell, George G Zhanel, Coleman Rotstein, John Muscedere, Mark Loeb, Jennie Johnstone

**Affiliations:** 1 Department of Medicine, McMaster University, Hamilton, Canada; 2 Department of Medical Microbiology and Infectious Diseases, University of Manitoba, Winnipeg, Canada; 3 Department of Medicine, University of Toronto, Toronto, Canada; 4 Department of Critical Care Medicine, Queens University, Kingston, Canada; 5 Pathology and Molecular Medicine, McMaster University, Hamilton, Canada; 6 Department of Laboratory Medicine and Pathobiology, University of Toronto, Toronto, Canada

**Keywords:** Canada, CAP, community, acquired pneumonia, COVID, 19

## Abstract

Dealing with coronavirus disease 2019 (COVID-19) has been a monumental test of medical skills and resources worldwide. The management of community-acquired pneumonia (CAP) can at times be difficult, but treating CAP in the setting of COVID-19 can be particularly trying and confusing and raises a number of challenging questions relating to etiology, diagnosis, and treatment. This article is based on the authors’ experiences and presents an overview of how CAP during COVID-19 is handled in Canada. We touch on the issues of microbial etiology in patients with CAP in the setting of COVID-19 as well as diagnostic, site of care, and treatment approaches. Published guidelines are the basis of management of CAP and are discussed in the context of Canadian data. We also outline the usual treatment approaches to COVID-19, particularly in patients who have been hospitalized.

## Teaser

This article represents the viewpoints of the authors on the management of community-acquired pneumonia in Canada during the COVID-19 pandemic. The issues, difficulties, and challenges pertaining to the etiology, diagnosis, and treatment of these patients are discussed.

Community-acquired pneumonia (CAP) is a significant cause of morbidity and mortality. Most cases are caused by relatively few pathogens, but recently severe acute respiratory syndrome coronavirus 2 (SARS-CoV-2) has been added to the list of “usual suspects.” The coronavirus disease 2019 (COVID-19) pandemic has had an immense and tragic medical, social, and economic impact and physicians must now consider it in their assessment of patients presenting with findings suggestive of CAP. The overlap of signs and symptoms in such patients makes it exceedingly difficult to determine the pathogen with certainty on initial assessment. Although guidelines exist to help with the management of CAP, we are far from understanding how to best handle it during this pandemic.

This article is not meant as an in-depth literature review but represents the viewpoints of the authors on CAP management in Canada during COVID-19. Hopefully this will help to provide some perspective on the treatment of this complex disease and may help in the development of treatment pathways.

## ETIOLOGY OF CAP DURING COVID-19

CAP is caused by both bacterial and viral pathogens that vary by site of care, immune status, and comorbidity [1]. *Streptococcus pneumoniae* remains the most common cause in hospitalized immunocompetent adults; other important bacterial pathogens include *Haemophilus influenzae*, *Staphylococcus aureus*, *Legionella pneumophila*, *Chlamydia pneumoniae*, *Mycoplasma pneumoniae*, and aerobic gram-negative bacilli [1]. Pre–COVID-19, influenza, respiratory syncytial virus, and human metapneumovirus were the most common respiratory viral pathogens identified in CAP patients [2]. The etiology remains unknown in approximately 50% of those hospitalized [1].

During the pandemic, hospitalization rates of CAP patients without COVID-19 declined in older adults [3]. Bacterial causes of CAP are not thought to have changed during the pandemic; however, CAP due to non–SARS-CoV-2 respiratory viruses has decreased [3, 4]. Within weeks of its emergence in 2020, SARS-CoV-2 almost completely replaced seasonally circulating respiratory viruses, suggesting the potential for competitive infection risk by SARS-CoV-2 [5]. Public health measures, including masking, hand washing, physical distancing, limiting close contacts, and quarantine, have also reduced the circulation of respiratory viruses, including influenza [3]. Hospitalization rates with CAP due to SARS-CoV-2 during the pandemic will depend on local epidemiology [6].

During influenza pandemics, rates of bacterial coinfection, particularly with *S pneumoniae*, were high, occurring in approximately 25% of critically ill patients [7, 8]. Based on retrospective studies, bacterial coinfection in hospitalized cases of confirmed SARS-CoV-2 pneumonia is relatively uncommon, occurring in approximately 3% of patients, with inconsistent pathogens reported [9, 10].

## DIAGNOSIS

In Canada, clinical criteria such as cough, dyspnea, and fever are used initially to make a presumptive diagnosis of CAP but confirmation requires a new chest radiographic infiltrate. In the prepandemic situation, patients seen by family physicians were infrequently sent for a chest radiograph. In more serious cases or with worsening, a radiograph may be requested. The benefit of radiography in this setting has not been proven, and Canadian guidelines do not recommend for or against an outpatient chest radiograph. However, with COVID-19, even with minor degrees of shortness of breath, the threshold for radiography is lower and patients are more likely to be referred for assessment to an emergency department. Typically, laboratory testing such as blood or sputum cultures is very limited in the outpatient setting.

In contrast, patients with suspected CAP, particularly those with moderate to severe symptoms presenting to emergency departments, are subject to a core set of investigations that we recommend. This includes chest radiograph; bloodwork including a complete blood cell count; biochemical tests; and, especially if likely to be admitted to hospital, blood and sputum cultures with antimicrobial susceptibility testing and testing for COVID-19 using reverse-transcription polymerase chain reaction (RT-PCR). With documentation of a specific pathogen such as methicillin-resistant *Staphylococcus aureus* (MRSA), defining the pathogen and its antimicrobial susceptibility pattern may allow de-escalation of treatment from broad-spectrum to directed therapy. Referring to sputum Gram stain and culture, the recent American Thoracic Society/Infectious Diseases Society of America (ATS/IDSA) guidelines state that “whether to culture patients or not should be determined by individual clinicians on the basis of clinical presentation, local etiologic considerations etc [11].”

In settings where testing of SARS-CoV-2 is done on platforms that test for other respiratory viruses, the findings should be carefully considered. If for example, influenza virus is found, specific antiviral treatment can be instituted. The presence of other respiratory viruses is in keeping with recent epidemiological studies that document the importance of primary viral infection as the main pathogen [12]. The possibility of a severe bacterial superinfection is increased in someone with a preceding respiratory viral infection compared to someone without such a prior infection [13].

For severely ill patients, urine for *Legionella* antigen and nasopharyngeal swabs for influenza should also be tested. If the initial COVID-19 test is negative but the patient is strongly suspected of having it, the test will usually be repeated. For patients hospitalized with pneumonia, testing sputum or other lower respiratory tract specimens (eg, endotracheal aspirates or bronchoalveolar lavage) by RT-PCR for SARS-CoV-2 is preferred.

## IMPACT OF ANTIMICROBIAL RESISTANCE

Current Canadian data describing antimicrobial activity for the most common bacterial CAP pathogens are available [14, 15]. Amoxicillin, amoxicillin-clavulanate, ceftriaxone and ceftobiprole, lefamulin, and respiratory fluoroquinolones (levofloxacin and moxifloxacin) are active against 98%–100% of *S pneumoniae* strains. Cefuroxime and doxycycline susceptibilities are approximately 94% and 85%, respectively, whereas macrolide susceptibility is only approximately 78%. Of the macrolide-nonsusceptible *S pneumoniae,* two-thirds demonstrate low-level resistance (minimum inhibitory concentration [MIC] = 1–8 µg/mL) while one-third demonstrate high-level (MIC ≥16 µg/mL) resistance. For community-associated MRSA, ceftobiprole, doxycycline, and lefamulin demonstrate the highest susceptibilities (≥94%), while macrolides (except *M pneumoniae* susceptibility at ~85%), doxycycline, lefamulin, and respiratory fluoroquinolones continue to maintain excellent activity against atypical respiratory pathogens (*C pneumoniae*, *M pneumoniae*, and *L pneumophila*) [14, 15].

## TREATMENT

Three antimicrobials have recently been approved for CAP treatment in Canada: intravenous (IV) ceftobiprole in 2018, IV amoxicillin-clavulanate in 2020, and IV and oral lefamulin in 2020 (approved but not yet available). Ceftobiprole has broad-spectrum activity including gram-negative bacilli (non–extended-spectrum β-lactamase–producing Enterobacterales as well as *Pseudomonas aeruginosa*), and gram-positive cocci including MRSA and penicillin-resistant *S pneumoniae* [16, 17]. Currently in Canada, ceftobiprole is primarily used as directed therapy for CAP patients with documented MRSA with or without bacteremia [18]. However, with its excellent activity against common bacterial CAP pathogens, ceftobiprole could also be considered for empirical use (along with a respiratory fluoroquinolone) to treat severe CAP patients with risk factors for either MRSA or *P aeruginosa* [19]. Combination therapy of IV amoxicillin-clavulanate plus a macrolide (eg, azithromycin) could be considered for hospitalized nonsevere CAP. This combination may provide a fluoroquinolone-sparing strategy. In 2 studies, IV/oral lefamulin has demonstrated clinical efficacy equal to respiratory fluoroquinolones for both outpatient and inpatient CAP treatment [20, 21]. Given its broad spectrum of activity, IV lefamulin may be considered in place of an IV β-lactam/macrolide combination or a fluoroquinolone in the setting of resistance, clinical failure, intolerance, or adverse effects with these regimens or as a fluoroquinolone-sparing regimen. Oral lefamulin may also be an option for patients in clinical settings with high rates of macrolide- and doxycycline-resistant *S pneumoniae*.

Because of the inability to rapidly distinguish bacterial from viral (including SARS-CoV-2) pneumonia, antimicrobial agents are frequently administered to COVID-19 patients for suspected bacterial coinfection. A meta-analysis of COVID-19 studies reported that although the estimated prevalence of bacterial coinfection was 8.6%, 75% of the patients were given antimicrobials, with the highest percentages administered to older and more severe patients [22]. This highlights the need for antimicrobial stewardship efforts to mitigate the impact of COVID-19 on unnecessary antimicrobial usage resulting in potential adverse events, *Clostridioides difficile* infection, and antimicrobial resistance.

In Canada, the site of care decision for CAP is usually based on an overall clinical impression rather than specific prediction rules. CURB-65 or the Pneumonia Severity Index criteria are generally employed only when the site of care decision is difficult or if few hospital beds are available. Once the decision is made, the specifics of antimicrobial therapy are addressed. In virtually all such situations, the physician initially does not know what the specific pathogen is and therefore initial treatment regimens are generally designed to provide empiric coverage for both typical and atypical pathogens. When considering CAP treatment in the setting of COVID-19, one needs to understand the treatment of “traditional” CAP vs COVID-19 pneumonia.

**Figure 1.**
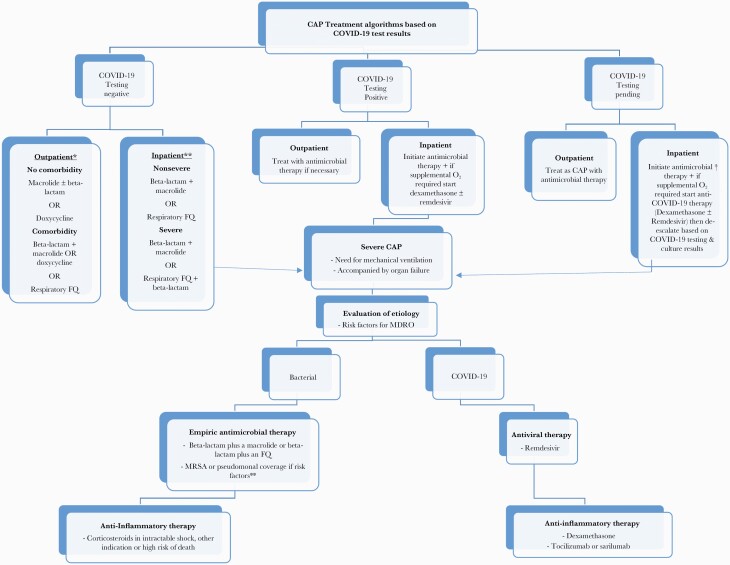
Community-acquired pneumonia treatment algorithms based on coronavirus disease 2019 test results. ∗If *Streptococcus pneumoniae* susceptibility to macrolides is >75% macrolide. ∗∗If risks for methicillin-resistant *Staphylococcus aureus* (MRSA) or *Pseudomonas aeruginosa*, add appropriate coverage to standard. †Treatment will depend on severity and risk factors for MRSA or *P aeruginosa*. Abbreviations: CAP, community-acquired pneumonia; COVID-19, coronavirus disease 2019; FQ, fluoroquinolone; MDRO, multidrug-resistant organism; O_2_, oxygen.

CAP treatment in Canada is generally very similar to that in the United States, and guidelines from both countries reflect this [1, 23]. The most recent ATS/IDSA guidelines reaffirm many of the previous recommendations but do not support macrolide monotherapy in outpatients if pneumococcal resistance to macrolides exceeds 25% [11]. Based on Canadian data, however (*S pneumoniae* 78% macrolide susceptible), macrolide monotherapy is still an option in milder cases (Figure 1).

## Outpatient Care of CAP

While guidelines from the United States and Canada provide information regarding types of antibiotics to be used, directives related to specific individual cases are not given, nor are they provided in this article. We urge physicians to consider each patient individually and to decide whether or not treatment is indicated based on such factors as severity, site of care, comorbidity, and risk of antimicrobial resistance. By its very definition, CAP is a syndrome with a compatible history and a new infiltrate on chest radiograph.

Outpatients are usually stratified into those without comorbidity or risk factors for antimicrobial resistance and those with comorbidity with or without such risk factors. If pneumococcal macrolide resistance exceeds 25%, treatment options often include a β-lactam such as amoxicillin plus azithromycin or doxycycline given alone. For patients in the comorbidity group, combination therapy with a β-lactam and either a macrolide or doxycycline may be used or monotherapy with a respiratory fluoroquinolone.

In cases of aspiration pneumonia, physicians often include anti-anaerobe coverage despite recent evidence indicating that generally such coverage is required only in cases with poor oral dentition, necrotizing pneumonia, or lung abscess, and we fully support this approach [24].

## Inpatient Care of CAP

Depending upon hospitalization on a medical ward or in an intensive care unit (ICU), treatment will differ. In both situations, however, initial therapy depends on whether or not the patient is at risk of infection with more resistant bacteria such as MRSA or *P aeruginosa*. For nonsevere cases initial treatment is usually with a combination of a β-lactam such as ceftriaxone plus a macrolide or monotherapy with a respiratory fluoroquinolone. If MRSA or *P aeruginosa* are of concern, physicians should modify the previous regimens to include appropriate coverage (Figure 1).

De-escalation in the form of narrowing the spectrum of treatment or discontinuation of antibiotic therapy should be based on laboratory data if available and clinical assessment. If a specific pathogen is identified, treatment may be narrowed to provide more selective coverage. Drugs may be discontinued in patients with proven bacterial infection or in those without a proven bacterial pathogen but who are COVID-19 negative and responding to antibacterial therapy. If the clinical course is uncomplicated, treatment may be stopped after 5–7 days. The same would apply to COVID-19, pending situations if the results come back negative and the patient is improving.

Antimicrobial stewardship is strongly promoted in Canada. Stewardship programs include leadership commitment, an evidence-based approach to antibiotic use, evaluation of such use, and encouragement of rapid de-escalation of antimicrobial therapy whenever possible. Monitoring prescription and resistance patterns by antimicrobial stewardship teams and education are also important. Hospitals are encouraged to provide local data on respiratory pathogens and their susceptibilities, and a national database is readily available as well. In keeping with recent CAP guidelines, markers such as procalcitonin are not used to make decisions regarding withholding of initial antibiotic treatment, nor are they generally used in Canada to indicate when to discontinue treatment [11].

A recent review highlighted a number of studies supporting a shorter duration of treatment such as 5–7 days if cases are uncomplicated and responding to treatment [25]. Unfortunately, therapy is often given for longer periods of time despite a lack of data to support this practice. Even in the outpatient setting, a recent study of 5.6 million outpatient antibiotic courses prescribed by 10 616 family physicians in Ontario, Canada, showed that the median treatment duration was 7–8 days and >30% exceeded 8 days. No significant differences in cure rates, however, were found in adults and children with a variety of infections including CAP [26].

Follow-up chest radiographs are usually done but often too early as it may require up to 8–12 weeks for resolution to occur. Unfortunately, the data on the utility of such imaging are sparse. We agree with the recent CAP guidelines that follow-up chest imaging is not necessary if CAP symptoms have resolved in 5–7 days.

## Treatment of Severe CAP

Severe CAP is defined as cases requiring mechanical ventilation or those with secondary organ failure such as septic shock [11]. Although scoring systems for early admission to ICU have been validated, they are infrequently used in Canada and, with limitations on ICU resources with COVID-19, the focus is on the delivery of adequate therapy irrespective of the setting. The 3 main principles for treatment of severe CAP are appropriate antimicrobial therapy, therapy for dysregulation of the inflammatory response, and support for secondary organ failure. These may vary depending on the presumptive or confirmed etiology emphasizing the importance of diagnostic testing.

Initial empiric antimicrobial therapy for severe CAP should include double coverage with a β-lactam plus a macrolide or β-lactam plus a fluoroquinolone [11] (Figure 1). Attention should be given to assessment of risk factors to help guide escalation of antimicrobial coverage beyond standard regimens if necessary. For example, is the patient immunocompetent and are there risk factors for antimicrobial resistance? Reevaluation is required for patients who worsen despite antimicrobial treatment and should include a reassessment of current antimicrobials and investigations to rule out complications such as lung abscess or empyema. In the presence of risk factors or progressive disease despite seemingly appropriate antimicrobial treatment, consideration should be given to broadening therapy to include resistant organisms such as MRSA and *P aeruginosa*. For COVID-19 patients, the risk of initial bacterial infection is low [22]. However, in the face of severe disease where there is uncertainty and bacterial coinfection is possible, most Canadian centers initiate empiric antimicrobials for CAP but de-escalate or discontinue them based on the results of bacterial cultures [27]. Empiric antiviral therapy for non–SARS-CoV-2 viruses is unnecessary. For severe CAP with proven influenza, however, oseltamivir is indicated [28]. There have been reports of prolonged viral shedding of influenza in critical illness and extended duration of therapy beyond the usual 5–10 days may be indicated, although the optimal duration is undefined.

## Treatment of Mild to Moderate COVID-19

The management of known COVID-19 cases depends upon the patient’s status. Patients may be asymptomatic or symptomatic and the latter group includes those with mild to moderate, severe, or critical illness based upon predefined criteria.

Asymptomatic and mild to moderate cases can be managed as outpatients. Asymptomatic infection does not require treatment, whereas select mild to moderate cases at risk of disease progression may receive monoclonal antibodies to prevent hospitalization. Formerly, bamlanivimab 700 mg IV within 10 days of symptom onset was found to decrease viral load but now is deemed ineffective against SARS-CoV-2 variants (Beta and Gamma) [29, 30]. However, casirivimab (1200 mg) plus imdevimab (1200 mg) as a single combined infusion or sotrovimab alone (500 mg IV) as a single infusion have recently been approved by Health Canada and provide effective treatment for COVID-19 [31, 32]. Emergency use authorization has been granted in the United States for casirivimab (600 mg)/imdevimab (600 mg) both by the IV and subcutaneous routes [33]. The study demonstrated similar efficacy for both the 1200 mg and 600 mg dosage regimens. The main impediment to the administration of these agents in Canada is a logistic one as establishment of outpatient infusion centers to administer the monoclonals has proven difficult.

Two other agents that have been reported to have a therapeutic effect in outpatient use are the antidepressant fluvoxamine and inhaled budesonide. Fluvoxamine has been reported to prevent clinical deterioration and reduce the need for emergency room visits and hospitalization [34]. For mild COVID-19, inhaled budesonide 800 µg twice a day for 14 days has demonstrated a reduced need for urgent care visits as well as improvement in time to recovery in 2 separate randomized clinical trials [35, 36]. Neither of these agents is licensed for such use in Canada.

Recently Merck and Pfizer released preprint information about interim analyses of their drugs, molnupiravir and paxlovid, respectively. The former is a nucleoside analogue and the latter a protease inhibitor. Paxlovid is given with ritonavir, which slows its breakdown, thereby presumably enhancing its effect. In randomized blinded controlled trials, both drugs appear to reduce the risk of hospitalization and death compared with placebo in patients with mild to moderate COVID-19 and with at least 1 risk factor for severe disease. As of this moment, monulpiravir has received approval in the United Kingdom. Neither of the medications has been approved in the United States or Canada at this point.

For suspected or proven COVID-19 infection, individuals meeting the criteria for severe infection generally require supplemental oxygen to maintain saturation at >94%. Once COVID-19 is confirmed, physicians may consider using prone positioning to optimize oxygenation [37]. Full therapeutic anticoagulation based on risk factors and potential benefit should be considered in patients with moderate to severe disease not requiring mechanical ventilation [38].

For patients requiring supplemental oxygen, dexamethasone 6 mg IV or orally or its equivalent dosing in other forms of corticosteroids for 10 days or until hospital discharge (whichever comes first) is strongly recommended as it reduces mortality in these patients [39, 40]. Remdesivir 200 mg IV initially followed by 100 mg daily for 4 days may shorten recovery time and can be used alone for those with lesser oxygen requirements who are not mechanically ventilated; however, it is in short supply in most Canadian hospitals [41]. Dexamethasone may also be combined with remdesivir.

## Treatment of Severe COVID-19

For severe COVID-19 pneumonia, remdesivir is the only approved antiviral drug, but there is conflicting evidence regarding its utility. The National Institutes of Health suggest its use in hospitalized patients requiring oxygen, both standard and high flow, but not those requiring invasive mechanical ventilation (IMV) [42]. The World Health Organization, however, has a conditional recommendation against its use in hospitalized patients, emphasizing the lack of conclusive evidence of efficacy [43]. Clinical trials continue and in the absence of definitive evidence, if remdesivir is available, we try to administer it as early as possible to patients requiring oxygen and continue it even if deterioration to IMV occurs.

The inflammatory response to CAP may lead to acute respiratory distress syndrome, septic shock, and multisystem organ failure. There is increasing evidence that the efficacy of anti-inflammatory therapy for severe CAP is dependent on the causal pathogen. For bacterial severe CAP, anti-inflammatory therapy is controversial and the best studied agents are the corticosteroids. Multiple meta-analyses yield conflicting conclusions, and recent guidelines recommended that such patients not receive corticosteroids routinely unless otherwise indicated or if there is refractory shock [11, 44].

While anti-inflammatory therapy is very important for COVID-19 pneumonia, there is weak evidence that corticosteroids may be harmful in influenza pneumonia [45]. For patients in whom both SARS-CoV-2 and influenza are isolated, there is no evidence to guide the clinician, but given the increased mortality with COVID-19 and the weak evidence of harm for influenza, steroid administration would be reasonable.

The mainstay of anti-inflammatory therapy for COVID-19 is corticosteroids since reduced mortality has been demonstrated [40]. The standard of care should be dexamethasone 6 mg/day; increased benefit with the drug has been noted with increasing disease severity [39]. Tocilizumab and sarilumab, both interleukin 6 receptor antagonists, have been reported to reduce mortality in COVID-19 with organ dysfunction in the ICU and should be administered with dexamethasone. Tocilizumab would be the first choice with sarilumab as an alternative if tocilizumab is not available [46]. The complex issue of trying to modulate the inflammatory response has been addressed recently by Rubin and colleagues [47]. Other anti-inflammatory agents for severe COVID-19 include baricitinib (a Janus kinase inhibitor) which in combination with remdesivir induced significantly faster recovery compared to remdesivir alone in patients receiving high-flow oxygen or noninvasive ventilation [48]. Recently, baricitinib when added to standard care and given at a dose of 4 mg/day for up to 14 days reduced 28-day mortality when compared with placebo in a randomized controlled trial [49]. However, baricitinib has not received Health Canada approval for the treatment of COVID-19.

Supportive therapy for severe CAP is as per the Surviving Sepsis Guidelines, but there are specific considerations depending on the most likely pathogen and infection control considerations [44]. For example, noninvasive ventilation with continuous positive airway pressure (CPAP) or bilevel positive airway pressure has been controversial for bacterial pneumonia due to potential inability to clear secretions, but there is less controversy with high-flow oxygen, which may provide better outcomes [50]. For influenza pneumonia, there have been reports that early use of CPAP and bilevel noninvasive ventilation leads to better outcomes and should be considered the first line of supportive care [51]. For COVID-19, high-flow oxygen is the first line of supportive therapy and infection control guidelines regarding the potential for aerosol generation should be followed. Additionally, in ICU patients, therapeutic dose anticoagulation did not improve survival compared with pharmacologic thromboprophylaxis [52].

Another issue to consider is the possibility of reactivation of latent tuberculosis either as a result of COVID-19 or its treatment with corticosteroids, which can result in immune dysregulation. Patients from foreign countries with high prevalence rates of tuberculosis and/or those with fibronodular findings on chest radiographs should have an interferon-γ release assay test done. In Canada only the QuantiFERON Gold test is available. This is particularly important given the multinational demographic of Canada, especially in densely populated cities.

## PREVENTION

Canadian authorities have generally mandated facial masking and social distancing to prevent COVID-19 infection while lockdown measures vary among provinces. Other supportive measures include the pneumococcal vaccine when indicated and the annual influenza vaccine.

The most important preventive measure, however, is the COVID-19 vaccine. Vaccine rollout in Canada has been successful in utilizing the messenger RNA and currently available viral vector vaccines [53]. More than 84% of the eligible population in the country has been doubly vaccinated. Ongoing vaccination efforts are focusing on the partially vaccinated and unvaccinated members of society. Future efforts will focus on administering a third vaccine dose to immunocompromised individuals as well as certain other members of society because of concerns of waning antibody levels and the ongoing risk of emerging SARS-CoV-2 variants.

Ultimately, the decision regarding the specifics of antimicrobial treatment of CAP in the setting of COVID-19 must be made on an individual basis. The physician must consider the clinical, epidemiologic, and diagnostic variables involved with each patient and be willing to reassess and modify therapy as necessary based on the clinical course of the patient and the results of diagnostic testing.

## Notes

***Patient consent.*** No individual patients or groups of patients were involved in any way or identified in the preparation of this manuscript. No specific patient consent was required. There was no human or animal experimentation and no approval was needed from local ethics committees to allow the writing of this manuscript.

***Potential conflicts of interest.*** All authors: No reported conflicts of interest.
